# Catastrophic Antiphospholipid Syndrome with Severe Acute Thrombotic Microangiopathy and Hemorrhagic Complications

**DOI:** 10.1155/2013/915309

**Published:** 2013-12-09

**Authors:** Gerardo B. Vieregge, Thomas J. Harrington, David M. Andrews, Maria F. Carpintero, Dollie F. Green, Ali Nayer

**Affiliations:** ^1^Department of Medicine, University of Miami, FL 33136, USA; ^2^Division of Hematology, University of Miami, FL 33136, USA; ^3^Department of Pathology, University of Miami, FL 33136, USA; ^4^Division of Rheumatology, University of Miami, FL 33136, USA; ^5^Division of Nephrology and Hypertension, University of Miami, Clinical Research Building, Suite 825, 1120 NW 14th Street, Miami, FL 33136, USA

## Abstract

The catastrophic antiphospholipid syndrome (CAPS) is a rare life-threatening form of the antiphospholipid syndrome characterized by disseminated vascular thrombosis resulting in multiorgan failure. On an exceedingly rare occasion, CAPS can be associated with severe hemorrhagic manifestations. We report a young woman with a history of several spontaneous miscarriages who presented with menorrhagia and hemoptysis. The patient developed respiratory failure due to diffuse alveolar hemorrhage. Laboratory tests demonstrated severe hemolytic anemia, profound thrombocytopenia, markedly elevated fibrin degradation products, and renal failure. Blood films revealed numerous schistocytes. Serologic tests disclosed hypocomplementemia and autoantibodies directed against several nuclear antigens. Coagulation studies revealed lupus anticoagulant. Echocardiography demonstrated reduced ejection fraction and moderate to severe mitral and tricuspid regurgitation. The patient was diagnosed with CAPS with hemorrhagic manifestations in the setting of new-onset SLE. The patient was treated with hemodialysis, high-dose glucocorticoids, plasma exchange, intravenous cyclophosphamide, and rituximab. Over the ensuing four weeks, the combination therapy led to hematological, cardiopulmonary, and renal recovery. This exceedingly rare case emphasizes that hemorrhagic manifestations, severe microangiopathic hemolytic anemia, and profound thrombocytopenia can dominate the clinical picture in CAPS.

## 1. Introduction

The antiphospholipid syndrome (APS) is an autoimmune disease characterized by vascular thrombosis, pregnancy complications, or both due to antiphospholipid antibodies [1, 2]. APS is due to pathogenic autoantibodies directed against proteins that interact with phospholipids. The disorder is referred to as primary when it occurs in the absence of another autoimmune disease. Secondary APS occurs in the setting of an autoimmune disorder such as systemic lupus erythematosus (SLE). Catastrophic APS (CAPS) is a rare life-threatening form of APS in which disseminated vascular thrombosis results in multiorgan failure [3–5]. On a rare occasion, CAPS is associated with hemorrhagic manifestations such as diffuse alveolar hemorrhage (DAH) [6, 7].

We report an exceedingly rare case of CAPS in the setting of new-onset SLE, in which the clinical picture was dominated by hemorrhagic manifestations including DAH and menorrhagia as well as severe microangiopathic hemolytic anemia and profound thrombocytopenia.

## 2. Case Presentation

A 32-year-old woman was transferred to our hospital for the management of severe acute thrombotic microangiopathy resulting in multiorgan failure.

The patient was in her usual state of health until she experienced heavy menstrual bleeding. Two days later, she developed fatigue and dyspnea on exertion while at work. She returned home and went to bed early to rest. Overnight, she developed cough, shortness of breath, chest pain, nausea, and vomiting. She was transported to a local hospital for evaluation. Her past medical history was notable for several spontaneous miscarriages and recent hair loss. She was a native of Haiti and worked in a local restaurant in Miami. She denied use of alcohol, tobacco, and drugs. On physical examination, the patient was a well-developed young woman in respiratory distress. The temperature was 37.0°C, the blood pressure 121/66 mm Hg, the pulse 106 beats/minute, the respiratory rate 42 breaths/minute, and the oxygen saturation 79% while breathing ambient air. Auscultation of the lungs demonstrated diffuse crackles. Chest radiograph revealed extensive bilateral pulmonary infiltrates. Supplemental oxygen was administered. However, the patient developed hemoptysis and was immediately intubated.

Laboratory data are summarized under “Day 1” in Table 1. There were severe hemolytic anemia, profound thrombocytopenia, and severe renal disease leading to azotemia, hyperkalemia, anion-gap metabolic acidosis, proteinuria, and hematuria. Blood films revealed numerous schistocytes. In addition, there were leukocytosis, neutrophilia, monocytosis, and hypoalbuminemia. Coagulation studies demonstrated markedly elevated D-Dimer. Erythrocyte sedimentation rate was elevated. Serologic tests disclosed antibodies against nuclear antigens including double-stranded DNA, chromatin, Smith antigen, ribonucleoproteins, SSA/Ro, and SSB/La. Complement C3 level was reduced and C4 was low-normal. Antibodies against glomerular basement membrane were not detected. Indirect immunofluorescence staining for antineutrophil cytoplasmic antibodies demonstrated a weak cytoplasmic pattern (c-ANCA). Plasma ADAMTS-13 activity (a disintegrin and metalloprotease, with thrombospondin-1—like domains) was normal. Serologic tests for hepatitis B and C and HIV were negative. Transthoracic echocardiography revealed reduced ejection fraction (45–50%) without regional wall motion abnormality as well as moderate to severe mitral and tricuspid regurgitation.

**Figure 1:**
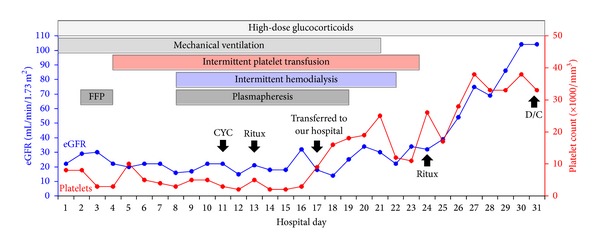
Renal function, platelet count, and therapeutic interventions during hospitalization. The estimate glomerular filtration rate (eGFR) is calculated using MDRD formula. Boxes represent the type and duration of specific treatments. See text for details. CYC: cyclophosphamide: D/C, discharged home; Ritux: rituximab.

The patient was diagnosed with severe acute thrombotic microangiopathy, DAH, renal failure, cardiomyopathy, and valvular heart disease in the setting of new-onset SLE. Over the ensuing two weeks, she was treated with high-dose glucocorticoids including intravenous methylprednisolone 1 g/day for three days, plasma exchange (a total of eight daily treatments), intravenous cyclophosphamide 1 g, and multiple blood transfusions (Figure 1). Hemodialysis was initiated for worsening renal function. Since no tangible recovery could be appreciated, intravenous rituximab (0.5 g) was administered. She was transferred to our hospital for further management after developing fevers and bleeding from gums and vagina.

Upon arrival at our hospital, the patient was sedated and intubated on mechanical ventilation. Bloody secretions were noted in the endotracheal tube. There were alopecia and hyperpigmented areas on the scalp. There were punctuate subconjunctival hemorrhage bilaterally and scattered petechiae on the lower extremities. Pitting edema was present over shins. Chest radiography revealed extensive bilateral pulmonary infiltrates. Renal ultrasonography revealed enlarged kidneys (13.2 cm in length) with increased echogenicity. There was no hydronephrosis, masses, or stones. Laboratory data are summarized under “Day 17” in Table 1. There were neutrophilia, lymphopenia, hemolytic anemia, profound thrombocytopenia, hypoalbuminemia, and renal insufficiency. Blood films revealed numerous schistocytes. Urinalysis revealed proteinuria and hematuria. Urinary protein excretion was estimated at 14.6 g/day based on a random urine protein to creatinine ratio. Urine sediment revealed numerous white blood cells and dysmorphic red blood cells. Several red blood cell casts and muddy brown casts were also observed. Serologic tests confirmed antinuclear antibodies of the same specificities previously reported. Complement C3 level remained low and C4 was normal. C-reactive protein was elevated. Plasma activity of ADAMTS-13 was normal. Antibodies against platelet factor-4, myeloperoxidase, proteinase-3, and glomerular basement membrane were not detected. Coagulation studies demonstrated markedly elevated D-Dimer. Elevated dilute Russell viper venom time (DRVVT) ratio and positive hexagonal phospholipid (Staclot LA) test indicated the presence of lupus anticoagulant.

The patient was diagnosed with catastrophic antiphospholipid syndrome with concurrent hemorrhagic manifestations in the setting of new-onset SLE. Plasma exchange therapy was continued with one plasma volume exchanged with fresh frozen plasma every day for three consecutive days. Intermittent hemodialysis was continued. Medications included prednisone 60 mg/day and hydroxychloroquine 400 mg/day. Platelets were infused as needed. On the 5th hospital day, the patient was extubated. In the meantime, urine output increased. On the 6th hospital day, the last hemodialysis treatment was given. On the 8th hospital day, intravenous rituximab 1 g was administered for persistent thrombocytopenia. A 24 hour urine collection demonstrated protein excretion of 12.7 g/day. On the 15th hospital day, the patient was ambulating requiring no supplemental oxygen. Laboratory tests revealed serum creatinine concentration of 0.8 mg/dL, hemoglobin concentration of 8.5 g/dL (no blood transfusion for 16 days), and platelet count 33,000/mm^3^ (no platelet transfusion for 7 days). She was discharged home on prednisone 60 mg/day and hydroxychloroquine 400 mg/day, dapsone for PCP prophylaxis, esomeprazole, and calcium/vitamin D supplements. She missed her follow-up appointments after discharge.

## 3. Discussion

We presented a young woman with a history of several spontaneous miscarriages who developed acute thrombotic microangiopathy associated with DAH, menorrhagia, renal failure, cardiomyopathy, and valvular heart disease. Laboratory tests revealed antinuclear antibodies and lupus anticoagulant. She was diagnosed with catastrophic antiphospholipid syndrome in the setting of new-onset SLE.

The catastrophic APS (CAPS) is a rare life-threatening form of APS in which disseminated vascular thrombosis results in multiorgan failure [3, 4]. The diagnosis of CAPS requires fulfillment of the following four criteria: (1) involvement of three or more organs/tissues; (2) development of manifestations in one week; (3) histological evidence of vascular thrombosis; and (4) presence of antiphospholipid antibodies on two occasions at least twelve weeks apart [5]. Our patient developed a catastrophic disease involving the lungs, kidneys, and heart in less than a week. Although we could not establish a histological diagnosis of vascular thrombosis due to severe thrombocytopenia, the presence of microangiopathic hemolytic anemia and markedly elevated fibrin degradation products including D-Dimer indicated vascular thrombosis.

In approximately one half of cases, CAPS is superimposed on APS [3, 4]. Although our patient had not been diagnosed with APS prior to her current presentation, a history of several spontaneous miscarriages could have been due to preceding APS. A concurrent autoimmune disorder is present in approximately one half of patients with CAPS. These include SLE (40%), lupus-like syndromes (5%), and other types of autoimmune diseases (9%). Although our patient did not have a diagnosis of an autoimmune disease, serologic studies at the time of current presentation revealed antibodies directed against several nuclear antigens. Antibodies against double-stranded DNA and Smith antigen are considered to be specific for SLE. CAPS affects predominantly women of childbearing age. A precipitating factor can be identified in the majority of patients. These include infection (22%), surgery (10%), discontinuation of anticoagulation (8%), medication (7%), obstetric complication (7%), and a neoplastic process (5%) [4]. We were unable to identify a precipitating factor in our patient.

The kidneys, lungs, central nervous system, heart, skin, liver, and gastrointestinal tract are most commonly affected [3, 4]. Pulmonary disease occurs in 24% of patients upon presentation and in 64% of patients during the course of the disease [4]. Acute respiratory distress syndrome and pulmonary embolism are the most common pulmonary manifestations. Thrombosis of pulmonary arteries and arterioles sometimes occurs. DAH occurs in 6% of patients [2, 6, 7]. The pathogenesis of DAH in CAPS is poorly understood. The histological examination of the lung in APS patients with DAH revealed microvascular thrombosis and, in some patients, capillaritis [6]. In our patient, hemoptysis and a characteristic chest radiograph suggested a diagnosis of hemorrhagic alveolitis in the context of CAPS. However, it is conceivable that severe thrombocytopenia accounted for or contributed to DAH. The absence of antiglomerular basement membrane antibodies at the time of presentation excluded Goodpasture's syndrome. Although indirect immunofluorescence staining for antineutrophil cytoplasmic antibodies demonstrated a weak cytoplasmic pattern, ELISA for antimyeloperoxidase and antiproteinase-3 antibodies was negative.

Present in only 18% of patients upon presentation, renal disease ultimately occurs in 71% of patients with CAPS [4]. The most frequent renal manifestations in CAPS are hypertension, proteinuria, hematuria, and acute renal failure [3, 4, 7–9]. Proteinuria averaged 2.8 g/day (0.6–6.1 g/day) [8]. Histologically, the most common finding is acute thrombotic microangiopathy characterized by deposition of fibrin thrombi in glomeruli, arterioles, or both. Interstitial inflammation is reported in one third of the patients. On a rare occasion, interstitial hemorrhage accompanies microvascular thrombosis [7, 9]. Immune-complex deposition is rarely observed. Interstitial fibrosis and tubular atrophy are indicative of chronic kidney injury. Concentric laminations of fibrotic intima of arteries and arterioles (onion skin pattern) indicate chronic vascular damage. In line with these observations, our patient presented with acute renal failure, nephrotic-range proteinuria, hematuria, and leukocyturia. Because of severe thrombocytopenia, renal biopsy was not performed. Nonetheless, heavy proteinuria in conjunction with dysmorphic red blood cells and red blood cell casts in the urine indicated severe glomerular injury.

Cardiac disease is present in 10% and 51% of CAPS patients upon presentation and during the course of the disease, respectively [3, 4]. Myocardial infarction, valvular heart disease, and arrhythmias are the most frequent cardiac manifestations and can result in heart failure and cardiac arrest. The following types of valvular heart disease were encountered in one third of the patients: mitral regurgitation (10%), tricuspid regurgitation (8%), Libman-Sacks endocarditis (6%), aortic regurgitation (4%), and combined mitral and aortic regurgitation (4%) [3]. Upon presentation, our patient was found to have moderate to severe mitral and tricuspid regurgitation as well as reduced ejection fraction without regional wall motion abnormalities. At the time of discharge, a follow-up echocardiogram revealed improved valvular function with only mild mitral and tricuspid regurgitation.

The clinical manifestations of CAPS are usually a consequence of acute thrombotic microangiopathy resulting in microangiopathic hemolytic anemia and thrombocytopenia. In approximately one half of CAPS patients, the platelet count is less than 100,000/mm [3, 4]. Thrombocytopenia in APS is believed to be due to antibodies against *β*_2_-glycoprotein-I causing platelet activation and aggregation as well as antibodies against platelet glycoproteins [10, 11]. The etiology of severe thrombocytopenia in our patient was likely multifactorial and included platelet aggregation and consumption due to severe vascular thrombosis and platelet loss due to DAH and vaginal bleeding. Because of the presence of several autoantibodies, it is conceivable that our patient could have had antibodies against platelet glycoproteins as well.

Microangiopathic hemolytic anemia and thromocytopenia can also be secondary to other types of acute thrombotic microangiopathies such as thrombotic thrombocytopenic purpura (TTP) and hemolytic-uremic syndrome (HUS) [12]. The differentiation between HUS/TTP and CAPS is sometimes difficult. As a general rule, schistocytosis are marked in HUS/TTP and mild in CAPS [12]. Fever and neurologic manifestations frequently dominate the clinical picture in patients with TTP [13]. In addition, plasma ADAMTS-13 activity is less than 5% of normal in most patients with TTP [13]. Our patient developed neither a fever nor neurologic manifestations upon presentation. In addition, her plasma ADAMTS-13 activity was normal. In the majority of patients with HUS, an infection with toxicogenic bacteria precedes the onset of the disease. No bacterial infection could be documented in our patient upon presentation. Furthermore, the presence of lupus anticoagulant and other autoantibodies was in favor of the diagnosis of CAPS in the setting of SLE.

Most CAPS patients are treated with anticoagulation and high-dose glucocorticoids [4]. However, additional therapeutic interventions such as plasma exchange, cyclophosphamide, intravenous immunoglobulins, and antiplatelet agents are often used in various combinations. Early diagnosis and treatment have reduced the mortality rate from 53% to 33% [14]. Considering severe thrombocytopenia and hemorrhagic manifestations, anticoagulation was not an option in our patient. However, a combination therapy consisting of high-dose glucocorticoids, plasma exchange, cyclophosphamide and rituximab resulted in hematological, cardiopulmonary, and renal recovery. In conclusion, this exceedingly rare case emphasizes that hemorrhagic manifestations, severe microangiopathic hemolytic anemia and severe thrombocytopenia can dominate the clinical picture in CAPS.

## Figures and Tables

**Table 1:** Laboratory data.

Analyte	Reference range	Day 1	Day 17
Sodium (mmol/liter)	137–145	137	141
Potassium (mmol/liter)	3.6–5.0	**5.9**	3.9
Chloride (mmol/liter)	98–107	107	105
CO_2_ (mmol/liter)	22–30	**11**	30
Urea nitrogen (mg/dL)	7–17	**33**	**88**
Creatinine (mg/dL)	0.5–1.4	**2.8**	**3.3**
Glucose (mg/dL)	74–199	**229**	145
Calcium (mg/dL)	8.4–10.2	**7.3**	8.7
Total protein (g/dL)	6.3–8.2	6.3	**5.5**
Albumin (g/dL	3.9–5.0	**1.8**	**2.9**
AST (IU/liter)	15–46	**58**	**70**
ALT (IU/liter)	9–52	23	30
LDH (IU/liter)	84–246	**836**	**5235**
Bilirubin, total (mg/dL)	0.3–1.3	0.4	**1.9**
Bilirubin, indirect (mg/dL)	0.2–0.9	0.3	**1.1**
Haptoglobin (mg/dL)	30–200	**8**	**<10**
Hemoglobin (g/dL)	12–16	**4.2**	**10.2**
Hematocrit (%)	37–47	**12.8**	**29.5**
Leukocyte count (×10^3^/*μ*L)	4.3–11.0	**25.3**	9.9
Neutrophils (×10^3^/*μ*L)	1.5–7.0	**18.5**	**9.3**
Lymphocytes (×10^3^/*μ*L)	1.0–3.7	3.3	**0.3**
Monocytes (×10^3^/*μ*L)	0.0–0.7	**1.3**	0.25
Eosinophils (×10^3^/*μ*L)	0.0–0.4	N/A	0.00
Basophils (×10^3^/*μ*L)	0.0–0.1	N/A	0.05
Platelet count (×10^3^/*μ*L)	140–440	**8**	**9**
Urinalysis			
Color	Yellow	Dark	**Red**
Turbidity	Clear	Cloudy	**Cloudy**
pH	4.6–8.0	5.0	6.0
Specific gravity	1.001–1.035	1.030	1.030
Glucose	Negative	Negative	**1+**
Ketones	Negative	Negative	Negative
Bilirubin	Negative	Negative	**1+**
Blood	Negative	**3+**	**3+**
Protein	Negative	**2+**	**3+**
Nitrites	Negative	Negative	Negative
Leukocyte esterase	Negative	**Positive**	**Positive**
White blood cells/hpf	0–2	**15**–**25**	**>182**
Red blood cells/hpf	0–2	**10**–**12**	**>182**
Urine protein (mg/dL)	5–25	**100**	**1197**
Urine creatinine (mg/dL)	20–370	N/A	82
Urine protein: creatinine	0.02–0.13	N/A	**14.6**
Lupus anticoagulant panel	Negative	N/A	**Positive**
Silica clotting test ratio	<1.2	N/A	<1.2
DRVVT ratio	<1.2	N/A	**1.21**
Staclot LA clotting time (sec)	<10	N/A	**12.2**
Anticardiolipin Ab	Negative	Negative	Negative
IgG (U/mL)	<10	0.4	4.1
IgM (U/mL)	<10	N/A	3.7
IgA (U/mL)	<8	7.4	1.4
Anti-*β*_2_-glycoprotein I Ab	Negative	Negative	Negative
IgG (U/mL)	<20	<9	<9
IgM (U/mL)	<20	<9	<9
ADAMTS-13 activity (%)	56–253	58	59
Antiplatelet factor-4	Negative	N/A	Negative
CRP (mg/dL)	<1.0	N/A	**5.3**
ESR (mm/hour)	0–20	**125**	N/A
Ferritin (ng/mL)	8–388	**397**	N/A
D-Dimer (ng/mL)	0–230	**4950**	**>5000**
FDP (*μ*g/mL)	<5	**5–20**	N/A
Fibrinogen (mg/dL)	190–380	344	228
APTT (sec)	24.5–35.7	23.2	21.3
PT (sec)	10.1–12.6	12.3	10.5
C3 (mg/dL)	90–180	**45**	**64**
C4 (mg/dL)	10–40	**9**	13
ANA titer	<1 : 40	**Positive**	**1 : 160**
Anti-dsDNA Ab (IU/mL)	<5.0	**85**	**10**
Antichromatin Ab (IU/mL)	≤0.9	**>8.0**	**Positive**
Anti-Smith Ab (IU/mL)	≤0.9	**>8.0**	**Positive**
Anti-RNP Ab (IU/mL)	≤0.9	**>8.0**	**Positive**
Anti-SSA/Ro Ab (IU/mL)	≤0.9	**>8.0**	**Positive**
Anti-SSB/La Ab (IU/mL)	≤0.9	**6.0**	**Positive**
Anti-Scl-70 Ab (IU/mL)	≤0.9	≤0.9	Negative
Anticentromere Ab (IU/mL)	≤0.9	≤0.9	Negative
Anti-Jo-1 IgG	Negative	Negative	Negative
Antiribosomal Ab	Negative	N/A	Negative
Anti-GBM Ab (AI)	<1.0	<1.0	<1.0
ANCA (perinuclear) (AI)	<1.0	<1.0	N/A
ANCA (cytoplasmic) (AI)	<1.0	**1.3**	N/A
Antimyeloperoxidase Ab	Negative	N/A	Negative
Antiproteinase-3 Ab	Negative	N/A	Negative
Rheumatoid factor (IU/mL)	<14	N/A	<14
CPK (IU/liter)	38–234	128	218
Alk Phos (IU/liter)	38–126	82	50

Values out of the reference range are in bold. Ab: antibody; ADAMTS-13: a disintegrin and metalloprotease with thrombospondin-1—like domains; AI: antibody index; Alk Phos: alkaline phosphatase; ALT: alanine transaminase; ANA: antinuclear antibodies; ANCA: antineutrophil cytoplasmic antibodies; anti-GBM: antiglomerular basement membrane; anti-dsDNA: antidouble stranded DNA; APTT: activated partial thromboplastin time; AST: aspartate transaminase; CO_2_: carbon dioxide, CPK: creatine phosphokinase; CRP: C-reactive protein; DRVVT: dilute Russell viper venom time; FDP: fibrin degradation products; LDH: lactate dehydrogenase; N/A: not available; PT: prothrombin time; RNP: ribonucleoproteins; Scl-70: scleroderma-70; sec: seconds.
